# ‘You can mix your methods, but you can’t mix your paradigms’: a guide to ontology and epistemology for the confused researcher

**DOI:** 10.3399/BJGP.2025.0390

**Published:** 2025-12-16

**Authors:** Stefanie Disbeschl, Katherine Checkland, Kris Stutchbury, Rebecca Payne

**Affiliations:** 1 North Wales Centre for Primary Care Research, North Wales Medical School, Bangor University, Bangor, UK; 2 Division of Population Health, Health Services Research and Primary Care, School of Health Sciences, University of Manchester, Manchester, UK; 3 Open University, Milton Keynes, UK; 4 Betsi Cadwaladr University Health Board, North Wales, UK; 5 Nuffield Department of Primary Health Care Sciences, University of Oxford, Oxford, UK

## Introduction

Research is an attempt to find out something useful about the world. Whether we like it or not, how we choose our methods, conduct data analysis, and draw conclusions flow from our underpinning assumptions about the nature of that world. In other words, all research is conducted from within a particular paradigm (Box 1). In this short article we aim to provide a brief summary or caricature, which will help the emerging researcher to develop confidence in the commonly used paradigms in primary care research, and to be better able to appreciate and appraise articles from paradigms outside their own.

Box 1. **Components of a paradigm**

A paradigm is a theoretical framework within which we make sense of the world. A paradigm is shaped by three components: ontology (What is the nature of reality? What exists?), epistemology (How do we know what our reality is? How do we know what we know?), and methodology (the way in which we find out knowledge — it is important to distinguish between methodology and methods here, where your methods are the actual tools you use to collect your data, the choice of which is influenced or determined by your methodology). These three pillars can be viewed as three questions to help us understand our paradigms, as posited by Guba and Lincoln:^1^ 1) What is the nature of reality? 2) What is the relationship between the knower and the known? 3) How can we come to know it?

## Why ontology and epistemology matter

Ontology and epistemology matter because we cannot begin to study the world until we have considered what we think we are looking at. In research as in life, our underlying assumptions about the nature of reality determine our actions. We know that this is true in real-life situations — for example, I believe that, if I step into a busy road, I will get run over, so I choose to use the method of looking both ways before stepping out. If I were to believe that magic is real and that I can use it to erect a forcefield around my body, I might behave differently. In research, the methods we choose to apply, and how we apply them directly, flow from the underlying assumptions that we make. Or, to put it more scientifically, the methods we choose are a direct consequence of the ‘paradigm’ (underlying philosophical assumption/definition of reality) within which we operate. These assumptions have implications for how we undertake, interpret, and use research.^1^

When researching the physical world, it is reasonable to expect that, under the same conditions, you would get the same results. If I apply a drug to a receptor in a laboratory under identical conditions, that receptor should respond in the same way every time. But if I then take that drug into a randomised controlled trial of people with depression, with a comparison group of people receiving talking therapies, how people respond to either therapy today might be completely different from how they respond in 6 months’ time, because of life events or social circumstances that sit outside the field of study.

*“Ontology and epistemology matter because we cannot begin to study the world until we have considered what we think we are looking at. In research as in life, our underlying assumptions about the nature of reality determine our actions.”*

Similarly, were I to develop a vaccine against a particular disease, in a laboratory situation I expect it to generate the same antibodies every time. But if I introduce it into a population, whether and how far it provides widespread population protection isn't governed by the same principles — media stories, people’s trust in healthcare providers, the extent to which vaccine conspiracy theories have traction within their social world, their ability to book vaccination appointments, all these determine whether adequate population coverage is achievable. Studying these factors involves making different assumptions from those of the laboratory world and requires different techniques.

These examples demonstrate that, where research involves social elements and human beings, a different set of assumptions and tools are required from those needed to study physical phenomena. Outside the wildest extremes of particle physics, the physical world has a demonstrable underlying reality that can generally be assumed to be stable and, at least approximately, knowable. The social world, by contrast, is forever in flux. Human beings respond and react in ways that are contingent and highly variable, not only varying between individuals but also varying over time — the same person may well respond differently to identical events at different times in their lives. How we understand and explain those differences will depend on our underlying understanding of the nature of the social world; in other words, our paradigm. Primary care research is quintessentially about people in particular social settings; it is therefore vital that researchers understand and can distinguish between the different paradigms.

## Major paradigms underlying healthcare research

Here, we outline four of the main paradigms underpinning healthcare research, namely *positivism*, *constructivism*/*interpretivism*, *critical realism*, and *pragmatism*. We will use the same example throughout to illustrate the differences between the four paradigms — the introduction of a new vaccine. Box 2 provides further information about each paradigm and the implications for research in health care.

**Box 2.** Summary of the different paradigms

Paradigm	Explanation	Implications	Types of methods consistent with it	Criteria to judge research
Positivism	There is a reality to be discovered — things are true or they aren’t true, they work or they don't work *Aim*: to find out the truth	Research wants to be as unbiased as possibleReduce the influence of the researcher on the findings/situationObjectivity prioritisedPositionality of the researcher irrelevant as the aim is to reduce the influence of the researcherRepeating the methods should ensure identical results, resulting in generalisability	Quantitative methodsQualitative methods that prioritise objectivity	Internal and externalvalidityReliabilityObjectivityReplicabilityRepeatability
Constructivism/interpretivism	There is no objective reality; reality is co-created by the people involved *Aim*: to create an account of a situation that can be used to better understand a phenomenon	Research findings are constructed by the interaction between researcher and researched — data emerge from the interaction between these individualsPositionality matters; swapping in a different researcher will lead to different results — this is OK but needs to be explicitly acknowledgedGeneralisablity beyond the specific circumstance rests on the use of theory Results may be transferrable to other settingsObjectivity is unattainable and undesirable	Mainly qualitative, all types	Validity reliabilityAcknowledgement of positionalityTrustworthinessCredibilityTransferabilityConfirmabilityTransparency — someone else can follow the steps you’ve made to come to your conclusions
Critical realism	There is a social reality ‘out there’ to be discovered, but our knowledge of it will never be complete. The things we observe and experience in the world are underpinned by ‘causal mechanisms’ and the purpose of the research is to bring to the surface those causal mechanisms. One of the purposes of critical realism research is to contribute to social theory	Critical realists recognise that we all come to understand the world in different ways. The findings of the research — the causal mechanisms — are arrived at through inference (abduction and retroduction). Evidence, theory, and context matter. The researcher is saying ‘this is how we think it is‘ but at the same time recognises that this might change if more evidence becomes available	Any methods or combination of methods that will enable you to answer the question (‘What is really going on here?’). Characterised by an in-depth study, for example, narrative interviews and multiple interviews with same participants. Methods are theory driven and interviews may involve testing theories through scenarios. Researchers often draw on evidence from a wide range of sources — the concept of an ‘open case study’ is helpful — and you don’t know at the outset where the evidence might come from	Authenticity — does it feel plausible to the people involved in the situation?Confirmability — is the evidence for the inferences convincing to the reader?Recoverability — is it clear how the researcher reached their conclusions? Is there a theoretical basis for the conclusions?
Pragmatism	Social reality is constantly evolving; humans assess and respond to it. The primary purpose of research is to get a solution that is useful	Less emphasis on the question of whether a hard social reality exists and more on 1) how that reality unfolds and is experienced; and 2) the real-world consequences of human decisions and actions	Any methods or combination of methods that help you answer the question	Usefulness and applicability in the real world

### Positivism

Within the positivist paradigm, reality is objective, can be measured, and exists independently of the researcher. Knowledge already exists, independently of the researcher, and positivists aim to uncover this knowledge, thus seeking ‘truth’ or ‘explanation’.

A positivist would likely be looking at the vaccine in terms of whether it is effective. This could be assessed through a randomised controlled trial (RCT), with control versus intervention participants. Data collected could include rates of infection, side effects, and effectiveness.

Because of its assumption of a single reality, positivism risks ignoring the human experience, meaning, and context, as it requires these to be eliminated as far as possible from the research situation.^1^ The focus on objectivity assumes that the researcher is completely neutral (and therefore unbiased). Although this may be routine in a laboratory study, it is very difficult to achieve in any research that depends on interactions between human beings.

### Constructivism/interpretivism

For the interpretivist, reality is subjectively experienced and understood.^2^ Researcher and participant together construct knowledge. This allows for the existence of multiple realities, rather than one single reality, as is the case in positivism.^3^ There is no ‘ultimate truth’^3^ but, rather, multiple versions of it depending on the interaction between researcher and participants. Researchers working within this paradigm focus on understanding (the meaning behind) human action, rather than causality and explanation.^4^

In the vaccination example, the interpretivist would be looking at how patients might feel about receiving the vaccine, or how they experience it. Data could be collected through in-depth interviews, or focus groups, and would focus on patients’ accounts, considering factors such as culture and stigma, for example. The data collected will be different according to the characteristics of the researcher involved, but, rather than attempting to minimise these differences, the research team openly acknowledge them. For example, researchers of different ages and professional backgrounds may elicit very different responses from participants. This is made explicit and seen as providing additional richness.

*“… different paradigms allow the researcher to address different types of research questions … in addition to determining the type of research question, the paradigm adopted will feed through into the way in which data are collected and analysed.”*

In contrast with positivism, where the aim is to provide an account that is as close as possible to ‘the truth’, the view that there are multiple realities means that a report of the findings from an interpretivist study must be explicit about whose perspective is being considered, as well as acknowledging the relevant contextual conditions.^3^

### Critical realism

Critical realists believe that there is an underlying reality, but it can only be partially known. Context plays an important role in understanding the social reality.

Critical realism is about looking for explanations (causal mechanisms) through a focus on what people are able to achieve (agency) in the social context in which they are operating (structures). It sees reality as being like an iceberg: most of reality (the iceberg) is invisible to the observer. The causal mechanisms exist below the surface, but give rise to ‘experiences’ and ‘events’, and these can be used to infer things about the underlying reality. Critical realism is ‘theory driven’. This means that it draws on a variety of social theories to seek explanations for observed phenomena. It assumes ‘judgemental rationality’, which allows researchers to compare and assess the explanatory power of different theoretical explanations and select the most appropriate explanation based on existing knowledge. The ‘critical’ in critical realism thus highlights the fact that researchers are required to be critical of the theories that they use and the explanations that they propose.

In the vaccine example, a critical realist might not only collect and examine quantitative data but also seek to understand the reasons why a vaccine was declined by a particular community through in-depth interviews seeking to understand previous experiences of health care, community experiences of authority such as interactions with healthcare professionals, and cultural views about medicines.

The focus on ‘structure’ and ‘agency’, and the stratified view of reality, provide a strong analytical framework for research in social contexts, which can be attractive to students. But the depth required often means that the sort of detailed data required are not always available, particularly to outsider researchers, and the ethical issues can be considerable as the research may unearth difficult conclusions. For example, if vaccine refusal has been driven by a community’s experience of state-directed healthworkers giving parenteral contraceptives without consent, the researcher faces a difficult decision as to what they should do with this information. Critical theory takes a similar ontological and epistemological stance to critical realism, but assumes that the underlying causal mechanisms in a given situation are linked to imbalances in power and marginalisation. A critical theorist investigating vaccine uptake would focus on marginalised communities with limited access to health care.

### Pragmatism

For the pragmatist, ‘reality is what works’ and is determined by human action and experience. Reality is viewed as flexible and context dependent; the researcher selects the perspective or approach that is most useful for addressing their research question. Pragmatism is often associated with the use of multiple methods, based on the belief that reality cannot be understood through one methodology alone.^5^

Using the vaccine example, a pragmatist would be interested in understanding how best to deliver an effective vaccine. This is likely to require both quantitative and qualitative data, with, for example, a quantitative study of vaccine uptake contextualised by qualitative data about why vaccines were accepted or refused.

Although proponents of pragmatism posit it as a paradigm, it is quite a different approach in comparison with the above paradigms, as it has less of a philosophical focus on the nature of reality and more of a focus on the purpose of the inquiry. Despite offering a flexible approach, there is a risk that the researcher may choose their research methods based on convenience and therefore the philosophical underpinnings may become blurred.^6^ This may have implications for the rigour of the study, as the criteria by which it should be judged may not be clear. The focus on practicality may also risk underplaying important ethical considerations such as equity and structural issues if the issues that surface during the study are unrelated to the purpose of the inquiry.

## Summary

These rather simplified accounts of the different paradigms highlight an important point: different paradigms allow the researcher to address different types of research questions. Furthermore, in addition to determining the type of research question, the paradigm adopted will feed through into the way in which data are collected and analysed. An interpretivist will look at interview transcripts in order to try to uncover the meanings underlying what the participants have said, whereas a critical realist will develop and test theories about the underlying causal mechanisms. Box 3 provides an example of how this might play out in practice. Importantly, as researchers, we can’t ignore paradigms; we have to choose. A positivist approach is vital for determining the technical efficacy of a vaccine, but if applied to a study of vaccine hesitancy would risk ignoring the important contextual issues at play.

Box 3. **Case study example**

A student undertakes a series of focus groups as part of public health research, undertaken within a positivist paradigm. When chairing a focus group, she accidentally shares her own views with participants. This supports them to open up, generating rich material and insights. When discussing with her supervisor, she is advised that she has introduced bias, and needs to discard these data. She writes up the study and includes in her limitations that she was the only researcher analysing the data, whereas best practice within the positivist tradition would have involved two separate researchers.

A student undertakes a series of focus groups as part of health services research, undertaken within a constructivist paradigm. When chairing a focus group, she chooses to share her own views with participants. This supports them to open up, generating rich material and insights. When discussing with her supervisor, she is congratulated for the richness of the insights gained. She reflects on her positionality as she writes up the study and includes all the material in the article.

### Common pitfalls

There are a number of epistemological traps waiting to ensnare the unwary researcher. Because everything else flows from the paradigm and the resulting epistemology (that is, the theory as to what it is possible to know in any given situation), it is important to be explicit and thoughtful about it during the design stage of a research study. As alluded to in the title of this article, methods can be mixed, but researchers need to be clear on the paradigm under which they are operating. For example, when analysing qualitative data, positivists will want two independent researchers analysing and comparing notes in order to ‘reduce bias’ and ‘get to the truth’. This contrasts with a constructivist paradigm, where it is the interaction of the researcher and the material that shapes the meaning, suggesting that the two independent coders are either unnecessary or viewed as adding depth rather than converging on a ‘truth’. Thus teams need to discuss the paradigm within which their research is to be conducted before it starts, so that methods are used consciously and in a consistent manner. It is a common misconception that qualitative methods are synonymous with an interpretivist paradigm; on the contrary, a positivist qualitative study is possible and potentially valuable, as long as the research question is appropriate to the paradigm.

When reading a research article, or undertaking peer review, it is important to recognise that what good looks like is defined differently for different paradigms. Quality from a positivist perspective is defined differently from quality within a critical realist perspective. Box 2 provides criteria by which research from each paradigm is judged, and it is important to bear these in mind when undertaking peer review. Many qualitative researchers bear the scars of being told that their research is ‘biased’ by a positivist colleague; in the spirit of team science, and acknowledging the richness and complexity of the situations we are researching, we owe it to one another to make the effort to understand these differences.

Finally, communicating with colleagues coming from different epistemological stances requires thoughtful consideration. Such colleagues may quite literally see the world in a different way. Being explicit about such differences will facilitate more effective communication and potentially allow for a shared understanding and a richer account of the problem being researched. Some researchers may resist this; it is easy, if you have always worked within a single paradigm, to fall into the trap of seeing your particular world view as ‘the way things are’. However, if we are to work together effectively to solve the wicked problems we face in society, we need all the paradigms we can muster.

### Conclusion

Wrestling with philosophy is outwith the comfort zone for many medical researchers; however, understanding the assumptions we bring to our research is vital in order to inform methods selection and generate credible research.

## Open access

This article is Open Access: CC BY 4.0 licence (http://creativecommons.org/licences/by/4.0/).
